# Dynamic Assessment of Water Quality Based on a Variable Fuzzy Pattern Recognition Model

**DOI:** 10.3390/ijerph120202230

**Published:** 2015-02-16

**Authors:** Shiguo Xu, Tianxiang Wang, Suduan Hu

**Affiliations:** Faculty of Infrastructure Engineering, School of Civil and Hydraulic Engineering, Dalian University of Technology, Dalian 116024, China; E-Mails: sgxu@dlut.edu.cn (S.X.); husuduan@mail.dlut.edu.cn (S.H.)

**Keywords:** water quality assessment, fuzzification, dynamic, interval influence, characteristic level value

## Abstract

Water quality assessment is an important foundation of water resource protection and is affected by many indicators. The dynamic and fuzzy changes of water quality lead to problems for proper assessment. This paper explores a method which is in accordance with the water quality changes. The proposed method is based on the variable fuzzy pattern recognition (VFPR) model and combines the analytic hierarchy process (AHP) model with the entropy weight (EW) method. The proposed method was applied to dynamically assess the water quality of Biliuhe Reservoir (Dailan, China). The results show that the water quality level is between levels 2 and 3 and worse in August or September, caused by the increasing water temperature and rainfall. Weights and methods are compared and random errors of the values of indicators are analyzed. It is concluded that the proposed method has advantages of dynamism, fuzzification and stability by considering the interval influence of multiple indicators and using the average level characteristic values of four models as results.

## 1. Introduction

Water is essential for peoples’ health and socioeconomic development. With the improvement of living standards, people have not only put efforts into safeguarding sufficient quantities of water resources, but have also paid more attention to increasing water quality. Water quality assessment can synthetically quantify the water quality state by choosing an appropriate assessment method and is gradually becoming an important tool for scientific utilization and management of water resources [1]. In recent years, comprehensive assessment of water quality has attracted a lot of interest. Changes of water quality state or level are driven by the interacting influences of physical, chemical and microbiological indexes. Complex and various indicators lead to imprecise comprehensive evaluations of water quality [2,3,4]. In addition, variations of external circumstances also result in the dynamic change of water quality. Seasonal variations change the water temperature and water surface conditions, and further influence the distribution of dissolved oxygen in depth; rainstorms bring lots of pollutants into the water, which might increases the total phosphorus content, and also changes the hydraulic conditions [5,6,7]. In order to reasonably assess water quality, researchers have developed a series of methods to study the state of water quality. Nazeer *et al*. used the water quality index (WQI) method to determine the water quality of Soan River, and the method was better used during the pre-monsoon season [1]. Talalaj used a modified WQI method to study the change in groundwater quality, and the results showed that the highest WQI value was recorded in summer, while the lowest was in March [2]. Researchers found that WQI method can expediently transform lots of water quality data into a comprehensive number to represent the water quality level [3], but some parameters in the index equations could influence the final score dramatically and even lead to wrong estimates [4]. Ren *et al*. used the fuzzy comprehensive assessment (FCA) method to assess the water quality and analyze the influence of human activities [5]. Yang *et al*. used the FCA method to assess the groundwater status [6]. Although the FCA method can simply obtain the water levels and solve the problem of the fuzzy boundaries [3,6], it treats the indicators and standards as points which might miss the information contained in the original data [7]. Uddameri *et al*. used the principal component analysis (PCA) method to assess groundwater quality [8]. Kumarasamy *et al.* used the PCA method to assess the water quality of the Tamiraparani River basin [9]. The PCA method can produce a comprehensive index instead of multidimensional variables, which is convenient for the analysis of water quality, but the method has some drawbacks which limit its application, for example, the index values should be compliance with Kaiser-Meyer-Olkin and Bartlett’s test [10]. More than that, the support vector machine (SVM), probabilistic neural network (PNN), k-nearest neighbor (KNN) and artificial neural networks (ANN) methods were also used to classify water quality [11,12,13,14,15]. These methods have different characteristics and contribute to studying the water quality recognition and classification; yet, there are still some debates on the best methods for the assessment of water quality.

The water quality state changes dynamically and is determined by indicators with imprecise levels [3]. Accurate assessment of water quality should be in accordance with the water characteristics including dynamic change and fuzzification, which causes more trouble for the assessment. There are methods for studying the the fuzzification and uncertainty of the water resources management, such as the Inexact Two-Stage Water Quality Management (ITWQM) Model, the Interval-Fuzzy De Novo Programming (IFDNP) method, FCA method and the Monte Carlo method [6,16,17,18], but few studies have focused on the dynamic changes of the water quality assessment. Actually, dynamic assessment of water quality aims to recognize a comprehensive water quality state which considers the information of all relevant indicators under successive temporal and spatial conditions. Then it can be converted for calculating the generalized distance between indicators and standards. However, most water quality assessments are discontinuous and treat the indicators as points (either or no) [6], which neglects the fact that indicators continuously and imprecisely belong to some standards. This paper aims to explore a method which can dynamically and successively assess water quality and provide a tool for improving the utilization and management of water resources.

## 2. Dynamic Water Quality Assessment Method

### 2.1. Dynamic Change and Fuzzification of Water Quality Assessment

Obviously, under the conditions of inflow of extraneous pollution and release of endogenous pollution, the change of water quality indicators is successive, which introduces dynamics and uncertainty into any comprehensive water quality assessment, so the dynamic changes of indicators and fuzzy membership degree for indexes belonging to each standard should be considered in the assessment of water quality. Fortunately, dynamic assessment makes the results more successive and accurate [19]. Typically, researchers have assessed water quality continuously to solve the temporal and spatial change of indicators’ continuities. An *et al*. used the fuzzy comprehensive assessment method to assess the water quality of Songhuajiang in 2010 from January to October, which showed that the water quality in June and October were better [20]. Kumarasamy *et al*. studied the water quality of the Tamiraparani River from July 2008 to June 2009, which indicated the change of water quality was mainly influenced by seasonal variation [9]. However, there are few studies on the fuzzy and successive relative membership degree for indexes belonging to standards. Firstly, considering the influence of a single indicator, this paper assumes the total nitrogen concentration of water is 0.9 mg/L and changes to 0.6 mg/L after a period of time. Although the total nitrogen concentration of water varies during different periods, the level of water quality is level 3 by comparison with the water quality standard (Table 1). Actually, the level of the latter is closer to level 2 compared with the water quality standard. Thus one can perform inaccurate and fuzzy estimation on levels of water quality by simple comparison. In addition, considering the influence of multiple indicators, this paper assumes the total nitrogen concentration of water is 0.9 mg/L and the total phosphorus concentration is 0.012 mg/L. It is easily seen that the levels of indicators are different, one is level 2 and the other is level 3 compared with the water quality standard. Multiple indicators lead to more confusion for comprehensive assessment of water quality. As mentioned above, the indicators change dynamically and may imprecisely belong to a certain standard, which leads to the same characteristics in water quality assessment.

### 2.2. Proposed Variable Fuzzy Pattern Recognition (VFPR) Model for Dynamic Assessment of Water Quality

Various indicators describe different aspects of the water quality state, so the recognition of the comprehensive water quality is dynamic and fuzzy. The variable fuzzy set (VFS) theory extended Zadeh’s fuzzy set theory to provide a continuous way to ascertain the membership degree and function, and also to effectively solve the problem of fuzzy boundaries [20,21,22,23]. This theory is in accordance with the dynamics and fuzzification of water quality assessment and provides an effective tool for complicated water quality evaluation issues [23,24], which is widely used in various kinds of assessments, such as agricultural drought risk assessment [21], water shortage risk assessment [7], and comprehensive risk evaluation for flood-control engineering systems [25]. It has been proved that the VFS theory can provide a stable method and make the results more reasonable due to the advantages of variable model parameters [7,24,25]. This paper explores a method to dynamically assess water quality based on the VFS theory.

**Table 1:** Indicator system and water quality standard.

Number	Indicators	Level				
		1	2	3	4	5
1	Dissolved oxygen (mg/L, X1)	7.5	6	5	3	2
2	Total nitrogen (mg/L, X2)	0.2	0.5	1	1.5	2
3	Total phosphorus (mg/L, X3)	0.01	0.025	0.05	0.1	0.2
4	Ammonia nitrogen (mg/L, X4)	0.15	0.5	1	1.5	2
5	*Coli* bacillus (/L, X5)	200	2000	10,000	20,000	40,000
6	Biochemical oxygen demand (BOD_5_) (mg/L, X6)	15	15	20	30	40
7	Chemical oxygen demand (COD_Mn_) (mg/L, X7)	2	4	6	10	15
8	Mercury ion (mg/L, X8)	0.00005	0.00005	0.0001	0.001	0.001

Because the water quality standard (GB3838-2002) is determinate, the assessment of water quality is changed by recognizing the comprehensive level of indicators. This paper assumes the set of samples of water quality is expressed as *X* = (*x_ij_*), where *i* = 1, 2, …, *n*, *n* is the total number of samples; *j* = 1, 2, .., *m*, *m* is the total number of indicators; and the set of standards of water quality is expressed as *Y* = (*y_hj_*) where *h* = 1, 2, .., *c*, *c* is the highest level of standard of the corresponding indicator *j*. In order to calculate a comprehensive level of indicators, a uniform format of the data set is necessary. Then the indicators (*x_ij_*) and standards (*y_hj_*) are normalized (*r_ij_*, *s_hj_*) to remove the influences of inverse indices and different dimensions by choosing different equations. The positive indices are ones that are positively correlated with water quality such as dissolved oxygen; oppositely, the inverse indices are those which are negatively correlated with water quality such as total nitrogen, total phosphorus, and biochemical oxygen demand:

(1) $$r_{ij}=\{\begin{array}{c} 0\\ \frac{y_{cj}-x_{ij}}{y_{cj}-y_{1j}}\\ 1 \end{array}\begin{array}{c} x_{ij}\leq y_{cj}(\text{positive}\;\text{index}),x_{ij}\geq y_{cj}(\text{inverse}\;\text{index})\\ \text{positive}\;\text{index}\;\text{or}\;\text{inverse}\;\text{index}\\ x_{ij}\geq y_{1j}(\text{positive}\;\text{index}),x_{ij}\leq y_{1j}(\text{inverse}\;\text{index}) \end{array}$$

(2) $$s_{hj}=\{\begin{array}{c} 0\\ \frac{y_{cj}-y_{hj}}{y_{cj}-y_{1j}}\\ 1 \end{array}\begin{array}{c} y_{hj}=y_{cj},\text{positive}\;\text{index}\;\text{or}\;\text{inverse}\;\text{index}\\ \text{positive}\;\text{index}\;\text{or}\;\text{inverse}\;\text{index}\\ y_{hj}=y_{1j},\text{positive}\;\text{index}\;\text{or}\;\text{inverse}\;\text{index} \end{array}$$

where *x_ij_* is the value of indicator *j* of the sample *i*, *i* is the number of samples and *j* is the number of indicators; *y_hj_* is the value that defines standard *h* of indicator *j*, where *h* = 1, 2 …, *c*, *c* represents the highest level of standard; *r_ij_* and *s_hj_* are the results of normalization of the indicators (*x_ij_*) and standards (*y_hj_*).

**Figure 1:**
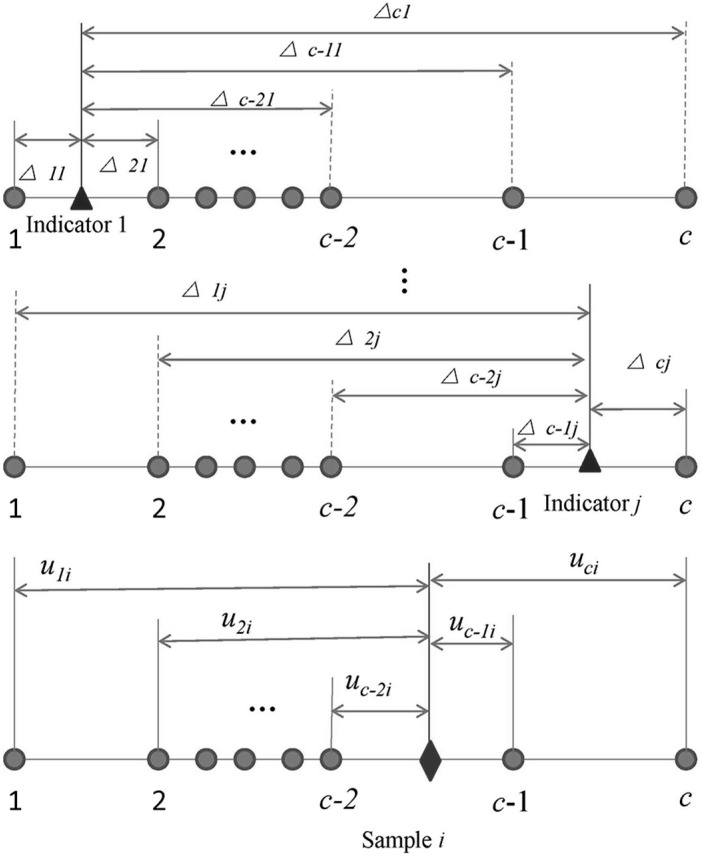
The recognition of the water quality level of sample *i* (△*_hj_* is the difference between indicator *j* of sample *i* and standard *h* of indicator *j*, *i* = 1, 2, …, *n*, *j* = 1, 2, …, *m*; *u_hi_* is the synthetic relative membership degree for sample *i* belonging to standard *h*, *h* = 1, 2, …, *c*).

This paper assumes sample *i* has *m* indicators, and the triangle, circle and rhombus respectively represent locations of indicator, standard and sample, which can be seen in Figure 1. It is easily found that the indicator 1 (triangle location) belongs to the interval between levels 1 and 2; the indicator *j* belongs to the interval between level *c*-1 and level *c*.

This scene results in fuzzification in the recognition of the level of sample *i* which may belong to arbitrary level from 1 (minimum level of sample *i*) to *c* (maximum level of sample *i*). Actually, there are differences between indicators and each standard (from level 1 to *c*), which can be expressed as △*_hj_* = *r_ij_* − *s_hj_*, where △*_hj_* is the difference between indicator *j* of sample *i* and standard *h* of indicator *j*.

These differences should be considered during the analysis of the level of sample *i* and can be converted to calculating the generalized distance by the Equation (3):

(3) $${\;}_{0}d_{hi}={\left[{\sum_{j=1}^{m}\left|r_{ij}-s_{hj}\right|}^{p}\right]}^{\frac{1}{p}}$$

where _0_*d_hi_* is the generalized distance between sample *i* and standard *h*, *h* is level of the standard; *h* = 1, 2, …, *c*; *p* is model parameter, *p* = 1 represents Hamming distance and *p* = 2 represents the Euclidean distance.

The term _0_*d_hi_* considers the successive differences between indicators of sample *i* and standards (from minimum level of sample *i* to maximum level of sample *i*) including information of the original data, and also can simulate different relationships between indicators with standards by changing the model parameter *p*. In addition, the indicators of water quality are playing different roles which can be expressed by different weights. Then, the weighted differences between sample *i* and each standard can be calculated by Equation (4):

(4) $$d_{hi}={\left\{\sum_{j=1}^{m}\left[w_{j}{\left|r_{ij}-s_{hj}\right|}^{p}\right]\right\}}^{\frac{1}{p}}$$

where *d_hi_* is the weighted generalized distance of indicator between sample *i* and corresponding standard *h*, *w_j_* is the weight of indicator *j*.

Next, *D_hi_* is established to solve the optimal synthetic relative membership u^*^_hi_, the weights w^*^ and center of clusters s^*^_hj_, which are based on *d_hi_* and weighted by *u_hi_*. Considering the general case, the weights w^*^ and center of clusters s^*^_hi_ are assumed to be unknown:

(5) $$D_{hi}=u_{hi}d_{hi}$$

where *D_hi_* is weighted generalized distance of sample including three variables (*u*, *s*, *w*), *u* is the synthetic relative membership degree, *s* is the center of the cluster, *w* is the weight of indicators.

Then, the objective function is as follows:

(6) $$min\left\{F\left(u,s,w\right)=\sum_{i=1}^{n}\sum_{h=1}^{c}u_{hi}^{2}d_{hi}^{a}=\sum_{i=1}^{n}\sum_{h=1}^{c}u_{hi}^{2}{\left[\sum_{j=1}^{m}w_{j}{\left|r_{ij}-s_{hj}\right|}^{p}\right]}^{\frac{a}{p}}\right\}$$

where *a* is the optimization criteria parameter to describe the relationship between indicator and standard, *a* = 1 (linear), *a* = 2 (nonlinear). Constraint conditions of Equation (6) are as follows:

(7) $$\sum_{h=1}^{c}u_{hi}=1,\forall i,\;0\leq u_{hi}\leq 1,\;\sum_{i=1}^{n}u_{hi}>0$$

(8) $$\sum_{j=1}^{m}w_{j}=1,\;0\leq w_{j}\leq 1$$

Lagrange function is established to solve the extremes of *u*, *s* and *w*:

(9) $$L\left(u_{hi},s_{hi},w_{j},\lambda_{u},\lambda_{w}\right)=\sum_{i=1}^{n}\sum_{h=1}^{c}u_{hi}^{2}{\left[\sum_{j=1}^{m}w_{j}{\left|r_{ij}-s_{hj}\right|}^{p}\right]}^{\frac{a}{p}}-\lambda_{u}\left(\sum_{h=1}^{c}u_{hi}-1\right)-\lambda_{w}\left(\sum_{j=1}^{m}w_{j}-1\right)$$

where $\lambda_{u}$ and $\lambda_{w}$ are Lagrangian multipliers of *u_hi_* and *w_j_* respectively.

Actually, for the water quality assessment, the center of clusters *s_hj_* is the water quality standard. The weight of indicator *w_j_* can be determined by lots of methods, such as the AHP model and the EW method. Then, *u_hi_* is solved as follows:

(10) $$u_{hi}=\{\begin{array}{l} 0\;,1\leq h\leq a_{i}\;or\;c\geq h\geq b_{i}\\ \frac{1}{\sum_{k=a_{i}}^{b_{i}}{\left[\frac{\sum_{j=1}^{m}{\left[\omega_{j}\left|r_{ij}-s_{hj}\right|\right]}^{p}}{\sum_{j=1}^{m}{\left[\omega_{j}\left|r_{ij}-s_{kj}\right|\right]}^{p}}\right]}^{\frac{a}{p}}},\;a_{i}\leq h\leq b_{i} \end{array}$$

where *u_hi_* is the synthetic relative membership degree for sample *i* belonging to standard *h*; *k* is the interval (*a_i_*, *b_i_*) to which sample *i* belongs; the *a_i_* and *b_i_* are obtained by comparing *r_ij_* with *s_hj_* (Figure 1), where *a_i_* is the minimum level of sample *i*, and *b_i_* is the maximum level of sample *i*; *m* is the total number of indicators; *w_j_* is the weight of the indicator *j*; *a* is the optimization criteria parameter, *a* = 1 (linear), *a* = 2 (nonlinear); *p* is the distance parameter, *p* =1 (Hamming distance), *p* =2 (Euclidean distance). *a* = 1, *p* = 1 expresses that the distance between indicator and standard is Hamming distance and the relation is linear; *a* = 2, *p* = 1 expresses that the distance between indicator and standard is Hamming distance and the relation is nonlinear; *a* = 2, *p* = 2, expresses that the distance between indicator and standard is Euclidean distance and the relation is nonlinear; *a* = 1, *p* = 2, expresses that the distance between indicator and standard is Euclidean distance and the relation is linear. The parameters (*a*, *p*) are changed to simulate unknown and different relationships between indicator and standard, which leads to stable results.

The traditional fuzzy assessment model often uses the maximum membership degree to determine the final level of sample, which neglects the information of other membership degrees. Fortunately, the VFPR model uses level characteristic values to determine the final level of sample, which contains successive information of relevant membership degrees and making the results more in line with the change of water quality:

(11) $$H=\sum_{h=1}^{c}u_{hi}h$$

where *h* is the level of standard, *h* = 1, 2… *c*, and *c* is the highest level of standard; *H* is level characteristic value of sample *i*.

### 2.3. Application Steps

The proposed model is a continuous way to dynamically and successively assess water quality, and the steps are as follows:

In the first step, the indicator system of water quality assessment should be developed following the principles of systematicness, causality and sustainability [26].

In the second step, Equations (1) and (2) are used to normalize (*r_ij_*, *s_hj_*) the indicators (*x_ij_*) and standards (*y_hj_*) so as to eliminate the influence of inverse indices and different dimensions respectively.

In the third step, the appropriate method for weighting indicators is selected. Generally, subjective weight can well reflect the opinions of researchers on the issue; oppositely, objective weight can better reserve the original data information adequately. This paper uses the AHP and EW method to determine subjective weight *w_1 (j)_* and objective weight *w_2(j)_* of indicator respectively, which have been successfully and widely used in a lot of assessments [27,28,29]. Then Equation (12) is used to determine the synthetic weight based on the two types of weights:

(12) $$w_{j}=\frac{w_{1(j)}\times w_{2(j)}}{\sum_{j=1}^{m}w_{1(j)}\times w_{2(j)}}$$

where *w_j_* is the synthetic weight of the indicator *j*, *w_1(j)_* is the subjective weight of the indicator, *w_2(j)_* is the objective weight of the indicator.

In the fourth step, the results of normalization *r_ij_*, *s_hj_*, synthetic weight *w_j_*, and interval (*a_i_*, *b_i_*) of sample *i* are put into Equation (10). Thus four results of *u_hi_* are calculated by changing the model parameters (*a* = 1, *p* = 1; *a* = 1, *p* = 2; *a* = 2, *p* = 1; *a* = 2, *p* = 2).

In the fifth step, Equation (11) is used to calculate the characteristic value *H* of the sample *i* based on the fourth step, then the average value is used as the assessment result.

### 2.4. Method Verification

Water quality assessment is a quantitative method for studying water quality state and changes. The assessment method and indicator system should reflect these characteristics reasonably. At present, eutrophication, heavy metals, microorganisms, and comprehensive pollution are the major pollution types in water [30]. In order to reflect comprehensive water quality reasonably, the indicators should represent all those aspects. The indicator system that doesn’t include all types of pollution would not realize that. This paper selects dissolved oxygen (DO), total nitrogen, total phosphorus, ammonia nitrogen, coli bacillus, biochemical oxygen demand (BOD_5_), chemical oxygen demand (COD_Mn_), and mercury ion to assess water quality. The indicator system covers aspects of eutrophication, heavy metal, microorganism, and comprehensive pollution which can describe water quality state overall. And the majority indicators are also used in the literature [3,20]. The water quality standard refers to the National Surface Water Quality Standard of China (GB3838-2002). The indicator system and standard are as follows.

As seen in Table 1, the five levels of water quality are: good (1), fine (2), ordinary (3), poor (4), and bad (5), while the water quality surpassing level 3 is considered suitable for drinking water supplies. Based on the water quality standard, 10 virtual water quality samples are created to verify the correctness of the proposed method. The samples and evaluation results can be seen in Table 2. The indicator values of Sample 1 are all better than level 1. Conversely, those of Sample 10 are worse than level 5. The indicator values of Sample 3 are just the average values of levels 1 and 2. Similarly, those of Samples 6, 8 and 9 are the average values of levels 2 and 3, leve1s 3 and 4, leve1s 4 and 5 respectively. Samples 2 and 4 are the same as Sample 3 except the dissolved oxygen (X1), of which the values are respectively closer to levels 1 and 2. Similarly, Samples 5 and 7 are the same as Sample 6 except the total nitrogen (X2). The total nitrogen value of Sample 5 is closer to level 2, while that of Sample 7 is closer to level 3.

The proposed method is used to assess the 10 virtual samples and the results can be seen in Table 2. The results are consistent with the original data information. Samples 2, 3 and 4 verify that the method can describe well changes of dissolved oxygen. Moreover, Samples 5, 6 and 7 show that the method can also describe well the changes of total nitrogen. Therefore, the proposed method can correctly assess water quality state and describe well changes of different indicators.

**Table 2:** Samples and results of assessment.

Samples	Indicators								Results (level)
	X1	X2	X3	X4	X5	X6	X7	X8	
1	8	0.1	0.005	0.075	100	7.5	1	0.000025	1
2	7.4	0.35	0.0175	0.325	1100	15	3	0.00005	1.19
3	6.75	0.35	0.0175	0.325	1100	15	3	0.00005	1.5
4	6.1	0.35	0.0175	0.325	1100	15	3	0.00005	1.81
5	5.5	0.51	0.0375	0.75	6000	17.5	5	0.000075	2.28
6	5.5	0.75	0.0375	0.75	6000	17.5	5	0.000075	2.5
7	5.5	0.9	0.0375	0.75	6000	17.5	5	0.000075	2.65
8	4	1.25	0.075	1.25	15,000	25	8	0.00055	3.5
9	2.5	1.75	0.15	1.75	30,000	35	12.5	0.001	4.5
10	1	3	0.3	3	50,000	50	20	0.002	5

## 3. Case study

This paper uses the proposed method to dynamically assess water quality of Biliuhe Reservoir, the important water source of Dalian city. The data sets are provided by the Biliuhe Reservoir Management Bureau of Dalian. In the first step, we develop the indicator system as mentioned in Section 1.4. In the second step, the Equations (1) and (2) are used to normalize the indicators (*x_ij_*) and standards (*y_hj_*), while dissolved oxygen is positive index which is positively correlated with water quality, the others are inverse indices negatively correlated with water quality. The results are as follows.

It is easy to observe in Table 3 the dynamic changes of the indicators. The majority of the indicators of water quality are better than level 2, but total nitrogen (X2) exceeds the standard of level 4 and dissolved oxygen (X1) in August and September is lower than the others. In the third step, the AHP model and EW method are combined to calculate the synthetic weights of the indicators. The subjective weight is determined by the AHP model, and the judgment matrix and weights are seen in Table 4, which refer to [17,20,21,25]. The objective weight is determined by the EW method and the detailed calculation process refers to [6,28]. Then two types of weights are used to calculate the synthetic weight by Equation (12).

**Table 3:** Results of normalization.

**Time**		**X1**	**X2**	**X3**	**X4**	**X5**	**X6**	**X7**	**X8**
Samples	200504	1	0.171	0.966	1	1	1	0.905	1
	200505	1	0	0.966	1	1	1	0.985	1
	200506	1	0	0.97	1	1	1	0.973	1
	200507	0.949	0	1	1	1	1	0.98	1
	200508	0.653	0	0.989	1	1	1	0.98	1
	200509	0.719	0	0.981	1	1	1	0.956	1
	200510	0.96	0	1	0.998	1	1	0.965	1
	200604	1	0	0.959	1	1	1	0.996	1
	200605	1	0	0.97	1	1	1	0.982	1
	200606	1	0	0.962	1	1	1	0.993	1
	200607	0.951	0	1	1	1	1	0.968	1
	200608	0.788	0	0.996	1	1	1	0.962	1
	200609	0.784	0	1	1	1	1	0.976	1
	200610	0.995	0	1	1	1	1	0.969	1
	200704	1	0.11	1	1	1	1	0.955	1
	200705	1	0	1	0.997	1	1	0.974	1
	200706	1	0	1	1	1	1	0.947	1
	200707	1	0	0.951	1	1	1	0.953	1
	200708	0.853	0	0.951	1	1	1	0.935	1
	200709	0.799	0	0.974	1	1	1	0.945	1
	200710	0.945	0	0.985	1	1	1	0.954	1
**Level**		**X1**	**X2**	**X3**	**X4**	**X5**	**X6**	**X7**	**X8**
Standard	1	1	1	1	1	1	1	1	1
	2	0.727	0.833	0.921	0.811	0.955	1	0.846	1
	3	0.545	0.556	0.789	0.541	0.754	0.8	0.692	0.947
	4	0.182	0.278	0.526	0.27	0.503	0.4	0.385	0
	5	0	0	0	0	0	0	0	0

Notes: 200504 means April of 2005 and the others are similarly defined.

**Table 4:** Judgment matrix.

Indicator	X1	X2	X3	X4	X5	X6	X7	X8
X1	1	0.5	1	3	2	3	3	2
X2	2	1	2	5	4	5	5	4
X3	1	0.5	1	3	2	3	3	2
X4	0.33	0.2	0.33	1	0.5	1	1	0.5
X5	0.5	0.25	0.5	2	1	2	2	1
X6	0.33	0.2	0.33	1	0.5	1	1	0.5
X7	0.33	0.2	0.33	1	0.5	1	1	0.5
X8	0.5	0.25	0.5	2	1	2	2	1

The largest eigenvalue of the matrix (Criteria) is 8.03; the consistency ratio is 0.003 < 0.1.

The synthetic weight (*w_j_*) includes the subjective weight and objective weight, which covers the opinion of researchers on the issue and the original data information (Table 5). In the fourth step, variables and parameters are put into Equation (10) and four results (*u_hi_*) are calculated by changing the model parameters (*a*, *p*). In the fifth step, Equation (11) is used to calculate the characteristic value *H* of sample *i* based on the fourth step, then use the average value as the assessment result. The results are shown below.

**Table 5:** The synthetic weight of indicator.

Weight	X1	X2	X3	X4	X5	X6	X7	X8
Subjective weight (AHP model)	0.168	0.316	0.168	0.053	0.094	0.053	0.053	0.094
Objective weight (EW method)	0.185	0.079	0.189	0.127	0.119	0.102	0.084	0.116
Synthetic weight (Equation (12))	0.246	0.197	0.251	0.053	0.088	0.043	0.035	0.086

As shown in Table 6, the water quality of Biliuhe Reservoir is between levels 2 and 3 and is suitable for drinking water supply during the study period. Moreover, the results are more stable by considering linear and nonlinear influences among the indicators. The rationality and dynamics of the proposed method are discussed in the next section.

**Table 6:** Results of dynamic assessment.

Sample	*a* = 1; *p* = 1	*a* = 2; *p* = 1	*a* = 2; *p* = 2	*a* = 1; *p* = 2	Average Level
200504	2.30	1.85	2.40	2.63	2.29
200505	2.36	1.92	2.53	2.72	2.38
200506	2.36	1.92	2.53	2.72	2.38
200507	2.38	1.95	2.55	2.72	2.40
200508	2.56	2.31	2.75	2.83	2.61
200509	2.51	2.23	2.71	2.81	2.57
200510	2.37	1.93	2.54	2.72	2.39
200604	2.37	1.93	2.53	2.72	2.39
200605	2.36	1.92	2.53	2.72	2.38
200606	2.37	1.93	2.53	2.72	2.39
200607	2.38	1.95	2.55	2.72	2.40
200608	2.47	2.15	2.65	2.78	2.51
200609	2.47	2.15	2.65	2.78	2.51
200610	2.35	1.88	2.52	2.71	2.36
200704	2.29	1.81	2.44	2.65	2.30
200705	2.34	1.87	2.52	2.71	2.36
200706	2.34	1.88	2.52	2.71	2.36
200707	2.38	1.95	2.54	2.72	2.40
200708	2.46	2.14	2.63	2.77	2.50
200709	2.48	2.17	2.66	2.78	2.52
200710	2.39	1.98	2.56	2.73	2.41

## 4. Results and Discussion

The results of the assessment should be in correspondence with the actual change process of water quality. This paper verifies the applicability of the proposed method by comparing with actual change of water and other assessment methods.

### 4.1. Analysis of Assessment Results

**Figure 2:**
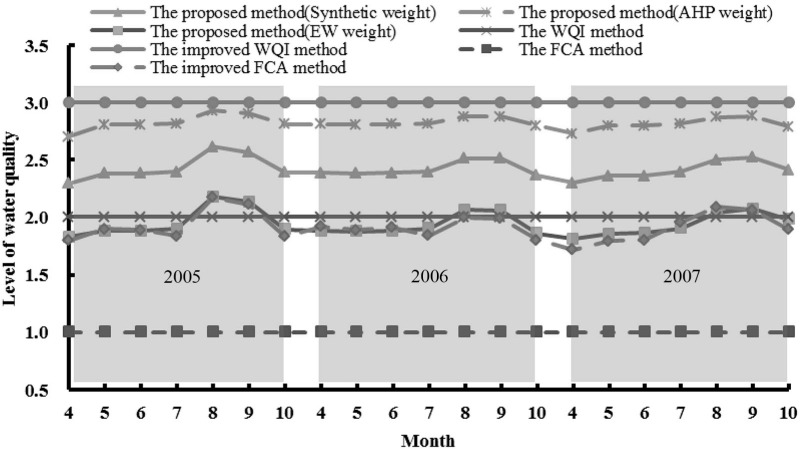
Results of water quality dynamic assessment.

Firstly, the results are compared with the actual status of water of the reservoir. Table 6, Figure 2 and Table A1 show that the overall, comprehensive water quality of Biliuhe Reservoir is suitable for drinking water supply. The levels of water quality are between levels 2 and 3 and have small changes monthly from April to October during the study period. The assessment results are consistent with the evolution of the reservoir environment and changes of the water quality indicators. Pollutant emissions have been controlled in the reservoir region and the forest coverage rate is 72.3% in the watershed, which further reduces the inflow of pollutants. In addition, most of the indicators are better than level 2. Only total nitrogen is worse than level 4, even level 5. Therefore, the levels of water quality of Biliuhe Reservoir assessed by the proposed method are reasonable under these conditions.

Figure 2 shows that although the change of the levels of water quality of the reservoir are small, the change trend in each year is similar. The level of comprehensive water quality of the reservoir decreases slightly from April, and reaches the minimum during August or September, and then it gradually improves from October. The annual monthly average water temperature of Biliuhe Reservoir is 24.1 °C in August and 22 °C in September, which are both higher than the others. Previous studies indicate that the water temperature is negatively correlated with dissolved oxygen content [31]. The higher water temperature in August and September impedes oxygen exchange between the atmosphere and water which leads to lower dissolved oxygen content (5.5–6.7 mg/L) compared with the other months (greater than 7.5 mg/L). Besides, the nonpoint pollutants are discharged into the reservoir during the summer, which has a negative effect on water quality. Thus, the water quality in August or September is the lowest under the dual pressures of high water temperature and heavy rainfall. The water temperature decreases continuously in October; accordingly, the dissolved oxygen content increases. The pollutants discharged into the reservoir are reduced after the flood season. External condition changes improve the water quality. Biliuhe Reservoir is ice-covered from November to next March. Winter processes increase dissolved oxygen content and conduce to deposition of internal pollutants, which improves the water quality in April. Similarly, the water quality declines gradually due to the increasing water temperature and rainfall from May to July, so the results and the trend are reasonable.

### 4.2. Comparison of Weights and Methods

Furthermore, the influence of different weights and methods is analyzed. First, the proposed method uses AHP weights, EW weights and the synthetic weight, respectively, to assess the same sample to compare the influence of weights. The results can be seen in Figure 2 and Table A1. It is easily found that the results of the synthetic weight version are between the AHP weight version and the EW weight version, and the results of three versions have the same trend. The results of AHP weight and EW weight versions are closer to levels 3 and 2, respectively. As mentioned in Section 4.1, most of the indicators are better than level 2 and only total nitrogen is worse than level 4, even level 5 in the Biliuhe Reservoir. The AHP weight considers the status of the serious total nitrogen pollution, which enhances the weight of total nitrogen and leads to worse water quality level. The EW weight is affected by the differences of the original data, which decreases the influences of those indicators with minor differences. Although the total nitrogen exceeds levels 4 or 5, its coefficient of variability (CV) is 19.8% and is lower than the average value (CV, 62%) of the eight indicators, which decreases the weight of total nitrogen and leads to better water quality level. The synthetic weight combines the two types of weight, which considers the subjective and objective influences and improves the drawbacks of using a single weight (subjective weight or objective weight). In the above section, we proved that the synthetic weight version is more reasonable to reflect the actual water quality in this case. Choices about how to weight the indicators can significantly influence the results of assessment. A situationally appropriate method should be used to determine the weights for different issues.

Then, the WQI method, the FCA method and their improved methods, which are widely used in the literature to analyze the influence of methods on the assessment results, are calculated [5,32]. As can be seen in Figure 2 and Table A1, the results of the proposed method are between levels 2 and 3 and have dynamic changes. The results in August and September are closer to level 3 and those in other seasons are closer to level 2. The results of the WQI method are between levels 2 and 3 too, which are level 2 under equal weight conditions and are level 3 under synthetic weight conditions (the improved WQI method). The equal weight way neglects the differences of indicators and reduces the impact of some important indicators, such as total nitrogen, which increases the water quality management risk. For this reason, the synthetic weight is more suitable for water quality assessment. The results of the FCA method are level 1. Then, the level characteristic value calculated by Equation (11) is used to substitute the maximum membership of the FCA method, and the results change and fluctuate around level 2 (the improved FCA method). The trend is similar to the proposed method. The results show that the WQI method and the traditional FCA method are defective when describing dynamic changes of water quality. Actually, the traditional FCA method treats the criteria or reference standards as points and only considers the influence of the adjacent-level of a single indicator, which misses information from the original data and might reflect the real water quality state imperfectly. Taking sample of 200708 as an example, while dissolved oxygen content is 6.69 mg/L, and total nitrogen content is 2.39 mg/L. The traditional FCA method considers dissolved oxygen belonging to levels 1 or 2; and total nitrogen content belonging to level 5 in the calculation process, but the differences between dissolved oxygen and levels 3, 4, 5; total nitrogen and levels 1, 2, 3, 4 are neglected in the traditional FCA method. Fortunately, the proposed method considers the interval influence of indicators, which calculates the differences between indicators and the continuous standard interval from the level 1 (minimum level of the sample) to 5 (maximum level of the sample). More than that, it is imperfect to use the maximum membership degree to determine the level of samples with the traditional fuzzy assessment model. Also taking the sample of 200708 as an example, the membership degrees of the sample in the five levels are 0.501, 0.303, 0, 0 and 0.197, respectively. According to the maximum membership degree principle (0.501), the sample is level 1. This produces confusion due to the fact the total nitrogen content is 2.39 mg/L, worse than level 5 (>2 mg/L). Actually, the total membership degree of other levels is 0.499 and has only a slight distinction with 0.501. Thus the missing information of other membership degrees may bring doubtful results. Then, Equation (11) is used to improve the FCA method, and gets the similar trend as the proposed method, so the proposed method can assess the water quality state reasonably and reflect its dynamic changes.

### 4.3. Uncertainty Analysis

Many uncertainties exist in the assessment of water quality. In the above section, this paper compares the influences of different methods and weights, but there is another uncertainty in the water quality assessment, which is from the original data. The variables inevitably have some random errors in the processes of monitoring and quantification [18,33,34,35,36,37]. The Monte Carlo method, the probabilistic point estimate methods (PEMs) and the perturbance moments method (PMM) are widely used in uncertainty analysis [18,38]. The Monte Carlo method is most suitable for unknown real-valued distributions and has been successfully used in [18,35]. Actually, the actual probability distribution of the index values is unknown.

This paper uses the Monte Carlo method to analyze the uncertainty of indicators, and the model parameters are as follows: the average μ is *x_ij_*; standard deviations σ are 0.1·*x_ij_*, 0.5·*x_ij_*, 0.9·*x_ij_* and 1.5·*x_ij_* respectively to analyze the influences of different degrees of deviation ; running times *N* is 1000. Then, the mean and 95% confidence interval of the *x_ij_* from Monte Carlo simulation are obtained.

**Figure 3:**
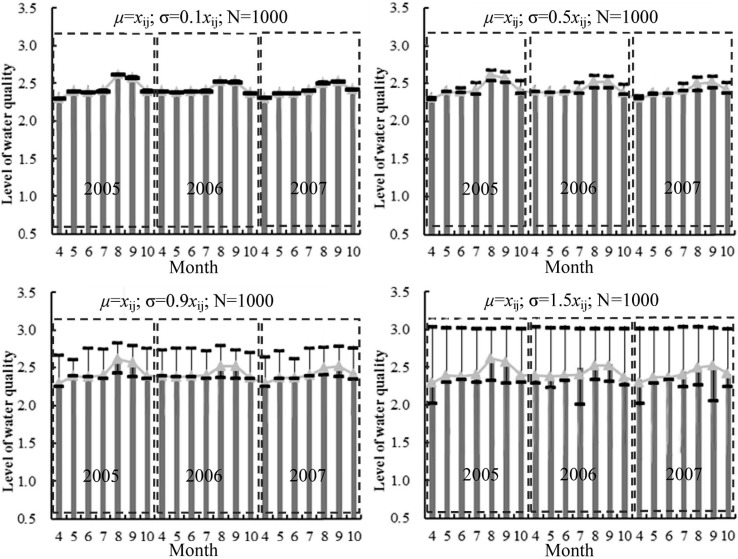
Uncertainty analysis showing the method results calculated using the proposed method (triangular gray dots), the mean from the Monte Carlo simulation (gray bars), 95% confidence interval (error bars). μ, σ and *N* are parameters of Monte Carlo model.

The results of the assessment can be seen in Figure 3 and Table A1. In general, the results of the actual and simulated samples are similar and both are located in the 95% confidence interval. The results of the simulation samples are similar to the actual samples (σ is 0.1 *x_ij_*), and there are small differences between the simulation samples and actual samples when σ is 0.5·*x_ij_* and 0.9·*x_ij_*. Until *σ* increases to 1.5·*x_ij_*, the lowest results of the simulation samples (Figure 3 and Table A1) change to level 3 which are different from the actual samples (between levels 2 and 3), so the proposed method has stable performance against random errors of indicator values. Furthermore, there are large uncertainties and deviations, and the higher the variance is, the more significant the deviation becomes.

## 5. Conclusions

This paper explores a method based on the VFPR model, and the proposed method is used to dynamically assess water quality. It considers the interval influence of multi-indicator and uses the average level characteristic value of four models as results, which is in accordance with the characteristics of fuzzification and dynamics of water quality. The synthetic weights of indicators are determined by the AHP model and EW method, which combine the advantages of subjective and objective weights. Then, the proposed method is used to assess the water quality of Biliuhe Reservoir. The results show that the water quality is between levels 2 and 3, and the water quality during August or September is worse than other months, which is in agreement with the changes of water temperature and rainfall. Comparison of the proposed method and other methods verifies that the proposed method is reasonable and is adept at describing dynamic change of water quality. The random errors of indicator value are analyzed using the Monte Carlo method; this shows that the random errors have impact on the results and the proposed method is stable. In addition, the flexible choice of the model parameters (*a*, *p*) for the assessment of water quality under different conditions is very important, and it will be discussed in a future study. Different uncertainty methods and probability distributions of indicator value should be further discussed to analyze the influence of the uncertainty of indicator values on assessment results. The authors believe that the proposed method contributes to the study of the dynamic changes of water quality, and could also provide a reference for water resource protection and similar studies.

## Appendix

**Table A1:** Detail results of different assessment methods and assessment of Monte Carlo simulation.

Samples	Methods							Assessment of Monte Carlo Simulation by the Proposed Method											
								*u* = *x*_ij_;σ = 0.1·*x*_ij_			*u* = *x*_ij_;σ = 0.5·*x*_ij_			*u* = *x*_ij_;σ = 0.9·*x*_ij_			*u* = *x*_ij_;σ = 1.5·*x*_ij_		
	①	②	③	④	⑤	⑥	⑦	mean	confidence interval		mean	confidence interval		mean	confidence interval		mean	confidence interval	
200504	2	3	1	1.80	2.70	1.83	2.29	2.30	2.30	2.30	2.30	2.28	2.31	2.30	2.25	2.66	2.31	2.02	3.02
200505	2	3	1	1.90	2.81	1.88	2.38	2.38	2.38	2.38	2.38	2.38	2.38	2.39	2.38	2.60	2.38	2.29	3.01
200506	2	3	1	1.89	2.81	1.88	2.38	2.38	2.38	2.38	2.38	2.38	2.44	2.38	2.38	2.76	2.38	2.33	3.01
200507	2	3	1	1.84	2.82	1.90	2.40	2.40	2.39	2.40	2.40	2.36	2.50	2.39	2.36	2.74	2.40	2.29	3.00
200508	2	3	1	2.17	2.93	2.18	2.61	2.62	2.61	2.62	2.60	2.53	2.68	2.63	2.42	2.82	2.57	2.32	3.00
200509	2	3	1	2.11	2.90	2.13	2.56	2.57	2.57	2.57	2.57	2.51	2.65	2.56	2.37	2.79	2.52	2.28	3.01
200510	2	3	1	1.83	2.81	1.89	2.39	2.39	2.39	2.40	2.43	2.36	2.53	2.40	2.36	2.76	2.36	2.30	3.00
200604	2	3	1	1.92	2.81	1.88	2.39	2.39	2.39	2.39	2.39	2.39	2.39	2.39	2.39	2.73	2.39	2.29	3.02
200605	2	3	1	1.89	2.81	1.88	2.38	2.38	2.38	2.38	2.38	2.38	2.38	2.38	2.38	2.75	2.38	2.22	3.02
200606	2	3	1	1.91	2.81	1.88	2.39	2.38	2.38	2.38	2.39	2.39	2.39	2.38	2.38	2.75	2.39	2.32	3.02
200607	2	3	1	1.84	2.82	1.90	2.40	2.39	2.39	2.40	2.40	2.36	2.50	2.41	2.36	2.72	2.48	2.00	3.00
200608	2	3	1	2.00	2.88	2.06	2.51	2.52	2.51	2.52	2.52	2.44	2.60	2.52	2.36	2.79	2.53	2.33	3.00
200609	2	3	1	1.99	2.87	2.06	2.51	2.51	2.51	2.52	2.51	2.43	2.59	2.47	2.36	2.73	2.53	2.30	3.00
200610	2	3	1	1.80	2.80	1.86	2.36	2.36	2.36	2.37	2.37	2.36	2.48	2.36	2.36	2.70	2.36	2.26	3.00
200704	2	3	1	1.72	2.73	1.81	2.30	2.30	2.30	2.30	2.31	2.30	2.33	2.30	2.24	2.64	2.33	2.01	3.00
200705	2	3	1	1.79	2.80	1.85	2.36	2.36	2.36	2.36	2.36	2.36	2.36	2.36	2.36	2.71	2.36	2.28	3.00
200706	2	3	1	1.80	2.80	1.86	2.36	2.36	2.36	2.36	2.36	2.36	2.36	2.36	2.36	2.61	2.36	2.33	3.00
200707	2	3	1	1.95	2.82	1.90	2.40	2.40	2.40	2.40	2.40	2.40	2.49	2.39	2.39	2.76	2.44	2.24	3.03
200708	2	3	1	2.09	2.87	2.04	2.50	2.50	2.50	2.50	2.49	2.40	2.57	2.50	2.40	2.77	2.51	2.26	3.03
200709	2	3	1	2.06	2.88	2.07	2.52	2.52	2.52	2.52	2.51	2.43	2.59	2.53	2.38	2.78	2.51	2.05	3.02
200710	2	3	1	1.89	2.79	1.98	2.41	2.41	2.41	2.42	2.41	2.37	2.51	2.42	2.34	2.76	2.41	2.23	3.01

Notes: ① are results of the WQI method, ② are results of the improved WQI method,③ are results of the FCA method., ④ are results of the improved FCA method, ⑤ are results of the proposed method (AHP weight), . ⑥are results of the proposed method (EW weight), ⑦are results of the proposed method (Synthetic weight).
